# Depressive Symptoms, Functional Status, and Cardiovascular Parameters in Patients with Major Depressive Disorder Undergoing Cardiac Rehabilitation While Treated with Vortioxetine: A Prospective Observational Study

**DOI:** 10.3390/jcm15041374

**Published:** 2026-02-10

**Authors:** Iván Hoditx Martín-Costa, Rafael Colman, Álvaro García-Amador, Cristian García-Caballero, Jerónimo Acosta-Rueda, Marta Fontán-Esteban, Yerika Martín-Quero

**Affiliations:** 1 Clínica Colman Especialidades Médicas, 11008 Cádiz, Spain; 2 MOVE-IT Research Group, Department of Physical Education, Faculty of Education Sciences, Instituto de Investigación e Innovación Biomédica de Cádiz (INiBICA), Universidad de Cádiz, 11519 Cádiz, Spain

**Keywords:** vortioxetine, major depressive disorder, cardiovascular disease, cardiac rehabilitation, depressive symptoms

## Abstract

**Background/Objectives:** Real-world evidence on the use of vortioxetine within cardiac rehabilitation (CR) programs among patients with major depressive disorder (MDD) remains limited. We aimed to describe the evolution of depressive symptoms, functional status, and selected cardiovascular and biometric parameters in patients with MDD undergoing CR while receiving vortioxetine in routine clinical practice. **Methods:** This was a 12-week, prospective, observational study conducted at Clínica Colman (Cádiz, Spain) between July 2022 and July 2024. Adults diagnosed with MDD who were undergoing CR and receiving vortioxetine as part of routine care were included. Depressive symptoms (Hamilton Depression Rating Scale, HAM-D) and functional impairment (Sheehan Disability Scale, SDS) were assessed at baseline and at weeks 3, 7, and 12. Cardiovascular and biometric parameters were measured at baseline and at week 12. Repeated-measures ANOVA and paired *t*-tests were used for statistical analysis. **Results:** Forty-nine patients were included (mean age 65.6 years; 41% women). Over the 12-week follow-up period, mean HAM-D and SDS scores decreased over time (both *p* < 0.001). Changes were also observed in VO_2_ max, body weight, body mass index, and waist circumference (all *p* < 0.05). Left ventricular ejection fraction, blood pressure, and QTc interval showed no relevant variation during follow-up. Mild adverse effects were reported in 6.1% of patients. **Conclusions:** In patients with MDD and undergoing CR while receiving vortioxetine, longitudinal changes were observed in psychological, functional, and selected cardiovascular measures. These real-world data describe clinical trajectories within integrated rehabilitation settings and provide hypothesis-generating evidence for future controlled studies.

## 1. Introduction

Major Depressive Disorder (MDD) and cardiovascular disease (CVD) frequently co-occur, leading to complex clinical presentations and poorer health outcomes [1]. The prevalence of MDD among patients with CVD is estimated to be approximately 18%, substantially higher than in the general population, and varies according to the specific cardiovascular condition and disease stage [1,2]. In this population, depression has been consistently associated with increased morbidity [3,4,5], reduced adherence to medical treatment [6,7,8,9], poorer participation in rehabilitation programs [10,11,12], and higher rates of recurrent cardiac events and mortality [3,13,14].

Contemporary clinical guidelines, including those issued by the European Society of Cardiology, emphasize the importance of an integrated approach to the identification and management of depression in patients with CVD [2,15,16,17,18]. These recommendations highlight the concurrent use of pharmacological treatment for depressive symptoms when clinically indicated, the promotion of physical activity, and participation in structured cardiac rehabilitation (CR) programs as part of comprehensive secondary prevention strategies [19,20,21,22,23,24,25]. However, in routine clinical practice, the implementation of these recommendations is often heterogeneous, and evidence informing how antidepressant treatment is selected and combined with CR remains limited, particularly outside controlled trial settings [2,20,26].

In addition, cognitive dysfunction, frequently observed in older adults with depression, has been associated with reduced motivation, impaired executive functioning, and lower adherence to complex therapeutic interventions, including structured exercise and CR programs [2,27,28]. Although exercise-based rehabilitation is strongly recommended for patients with CVD and comorbid depression, there is currently no clear consensus on how depressive symptoms should be addressed within rehabilitation settings, nor on how pharmacological and non-pharmacological approaches are combined in everyday clinical care [2,25,29].

Despite these guideline recommendations, clinical decision-making in patients with CVD and comorbid depression is often challenged by the limited availability of real-world data describing antidepressant use and clinical trajectories in routine care settings [30,31,32,33,34]. Selective serotonin reuptake inhibitors (SSRIs) are frequently prescribed in this population; however, their use in older or polymedicated patients has prompted ongoing discussion regarding cardiovascular tolerability, including potential effects on cardiac conduction and hemodynamic parameters [19,20,35].

In this context, vortioxetine is a multimodal antidepressant that is increasingly used in clinical practice for the treatment of major depressive disorder, including in patients with medical comorbidities [20,36,37]. Previous clinical studies conducted in non-cardiac or mixed populations have reported improvements in depressive symptoms and cognitive domains, alongside a generally favorable tolerability profile [36,38]. However, data describing its use and associated clinical trajectories in patients with CVD undergoing structured CR programs are limited, particularly in real-world, non-interventional settings [2,20,39].

Previous randomized trials assessing interventions for depression in cardiac populations, such as MIND-IT and ENRICHD, have reported modest reductions in depressive symptoms, with limited and inconsistent effects on functional or cardiovascular outcomes [40,41]. Together with subsequent meta-analyses, these findings illustrate the complexity of managing depression in patients with CVD and the challenges of translating improvements in mood into broader functional or cardiovascular benefits [39,42].

In this context, observational data derived from routine clinical practice may provide complementary insights into how depressive symptoms, functional status, and cardiovascular parameters evolve over time in patients receiving integrated, multidisciplinary care, without implying treatment efficacy.

Within this framework, the present prospective observational study, conducted within the EMDCAR.OPS program, aimed to describe the clinical evolution of patients with MDD and coexisting CVD undergoing CR while receiving vortioxetine as part of routine clinical care. By characterizing longitudinal changes in depressive symptoms, functional status, and selected biometric and cardiovascular parameters over a 12-week follow-up period, this study seeks to provide exploratory, real-world descriptive data on observed clinical trajectories in an integrated pharmacological and exercise-based rehabilitation setting.

## 2. Materials and Methods

### 2.1. Study Design and Setting

This was a 12-week, prospective, non-interventional, observational, single-center study conducted at Clínica Colman (Cádiz, Spain), within the EMDCAR.OPS framework, aimed at assessing the evolution of depressive symptoms, functional status, and biometric and cardiovascular parameters in patients with MDD and CVD enrolled in a CR program under routine clinical care. As an observational study, analyses describe longitudinal trajectories during concurrent standard care and do not support causal attribution of observed changes to vortioxetine versus CR or other factors.

#### Representativeness and Size of the Study

For the sample size calculation, prevalence data for CVD in Spain were considered, estimated at 7.4% of the general population [43]. Based on national population figures for Spain in 2021 (47,344,649 inhabitants) [44], this corresponded to an estimated 3,503,504 individuals with CVD.

Taking into account that the prevalence of depression among individuals with CVD has been reported to be approximately 27% [45]; the estimated population of patients with both CVD and depression was 700,700, which constituted the reference population for the sample size estimation.

Based on this reference population, the sample size calculation was performed using a 95% confidence level and a standard margin of error of 15%, resulting in an estimated sample size of 44 subjects. The relatively wide margin of error was predefined in accordance with the limited recruitment capacity inherent to a single-center, real-world, non-interventional study.

### 2.2. Participants

Eligible participants were adults (≥18 years) diagnosed with MDD according to DSM-5 criteria [46], with a baseline score ≥ 14 on the 17-item Hamilton Depression Rating Scale (HAM-D) [47,48], documented CVD, and active enrollment in a phase II CR program between July 2022 and July 2024 at Clínica Colman (Cádiz, Spain).

Inclusion criteria were: (i) age ≥ 18 years; (ii) diagnosis of MDD according to DSM-5 criteria; (iii) baseline HAM-D score ≥ 14; (iv) presence of at least one cardiovascular diagnosis eligible for CR; and (v) provision of written informed consent.

Exclusion criteria included: (i) contraindications to treatment with novel antidepressants; (ii) use of antidepressant medication other than the novel antidepressant under observation; and (iii) any medical or clinical condition that, in the opinion of the treating physician, precluded safe participation in the CR program.

Participants were recruited consecutively from a single center as part of routine clinical practice. Treatment allocation was not randomized, and no control or comparator group was included, in accordance with the non-interventional observational design of the study.

Participants who did not complete the CR program were excluded from the final analysis.

### 2.3. Cardiac Rehabilitation Program

Participants were enrolled in a structured, supervised phase II CR (CR) program delivered as part of routine clinical care. Prior to program initiation, cardiovascular risk stratification was performed by a cardiologist. A baseline physical and functional assessment was jointly conducted by a rehabilitation physician and a physiotherapist to individualize exercise prescription.

The CR program consisted of 36 supervised sessions, performed three times per week, with each session lasting approximately 60 min. Sessions followed a standardized structure and were supervised at all times by a physiotherapist and a nurse, ensuring both exercise guidance and clinical safety.

Each session began with a brief warm-up focused on joint mobility and low-intensity movements, followed by resistance training performed in a circuit format targeting major muscle groups. The resistance training component was systematically structured using a medium-term periodization model, comprising a single macrocyle divided into three consecutive mesocycles of equal duration. The first mesocycle emphasized neuromuscular and cardiovascular adaptation using low intensity and volume with longer recovery periods. The second mesocycle applied an undulating load approach, alternating higher-volume or higher-intensity sessions with recovery-oriented sessions. The final mesocycle focused on consolidation of adaptations, prioritizing movement quality, regulation of perceived exertion, and functional transfer to activities of daily living.

Following resistance training, aerobic exercise was performed for approximately 20–30 min using either a treadmill (RAM 870 A, Sanro Electromedicina, Madrid, Spain) or a cycle ergometer (ergoselect 50, ergoline GmbH, Bitz, Germany), depending on patient characteristics and preferences. Aerobic training intensity and duration were progressively adjusted according to individual tolerance and cardiovascular response. Periodically, sessions dedicated to flexibility and mobility exercises were included.

Throughout all sessions, continuous ECG monitoring was performed using a telemetry system (ERS ECG-Telemetry, ERS-2, ergoline GmbH, Bitz, Germany). Heart rate and cardiac rhythm were monitored in real time, allowing immediate adjustment of exercise intensity and ensuring patient safety. In selected cases, outdoor training sessions were monitored (ERS-2, EOA system, ergoline GmbH, Bitz, Germany).

### 2.4. Intervention and Clinical Care

Antidepressant treatment was prescribed as part of routine clinical practice and was not assigned by the study protocol. Treatment decisions were made by the treating psychiatrist prior to study inclusion, based on clinical judgment and baseline symptom severity.

Although the EMDCAR.OPS protocol contemplated the use of different novel antidepressants, all patients included in the present analysis received vortioxetine, reflecting prescribing patterns at the study center during the recruitment period. Dose initiation and adjustments were performed according to routine clinical practice.

Concomitant psychotropic and cardiological medications were permitted and managed according to standard care. The study protocol did not mandate changes to cardiovascular treatment or to the CR program.

### 2.5. Assessments

Depressive symptom severity was assessed using the 17-item Hamilton Depression Rating Scale (HAM-D), a clinician-administered instrument widely used in clinical research to evaluate depression severity, with total scores ranging from 0 to 52 and higher scores indicating more severe depressive symptoms [47].

Functional impairment was evaluated with the Sheehan Disability Scale (SDS) a self-reported measure assessing impairment in work/school, social life, and family life. Each domain is rated on a 0–10 scale, yielding a total score ranging from 0 to 30, with higher scores reflecting greater functional impairment [49,50].

Anthropometric measurements included body weight, height, body mass index (BMI), and waist circumference. Body weight and height were measured using a calibrated digital scale with an integrated stadiometer (GIMA 27288 Pegaso, GIMA, Gessate, Italy), and BMI was calculated as weight divided by height squared (kg/m^2^). Waist circumference was measured with a flexible tape at the midpoint between the lower costal margin (10th rib) and the iliac crest, with the participant standing in a relaxed position, at the end of a normal expiration.

Blood pressure was measured manually by a single trained nurse using a standard sphygmomanometer. Measurements were obtained from the left arm with the participant in a seated position after a resting period of at least 5 min. For each assessment, the maximum blood pressure value recorded during follow-up was considered for analysis.

Cardiovascular assessments included resting electrocardiography, cardiopulmonary exercise testing, and transthoracic echocardiography. Resting electrocardiograms were obtained using a standard 12-lead electrocardiograph (MortaraELI 230 system, Mortara Instrument, Milwaukee, WI, USA), and the corrected QT interval (QTc) was calculated using the Bazett correction formula. Cardiopulmonary exercise testing with breath-by-breath gas analysis was performed on a treadmill using an incremental ramp protocol (ERGOSTIK-XSCRIBE system with ERGOCAR gas analyzer, ergoline GmbH, Bitz, Germany), and peak oxygen uptake (VO_2_ peak) was defined as the highest oxygen consumption achieved during exercise under maximal effort criteria. Transthoracic echocardiography was performed using a high-resolution ultrasound system (ACUSON NX3 Elite, Siemens Medical Solutions USA, Inc., Mountain View, CA, USA), and left ventricular ejection fraction was calculated using the biplane Simpson method. All cardiovascular examinations were performed and interpreted by the same cardiologist.

All assessments were performed at baseline and at the end of the 12-week follow-up period, in accordance with routine clinical practice.

### 2.6. Statistical Analysis

Descriptive statistics were used to summarize the sample characteristics. Changes in depressive symptoms and functional impairment, assessed using the HAM-D and the SDS, were analyzed using repeated-measures analysis of variance (ANOVA). When the assumption of sphericity was not met, Greenhouse–Geisser correction was applied. Post hoc pairwise comparisons were adjusted for multiple testing using the Bonferroni method. Residual normality was assessed using the Shapiro–Wilk test. As a sensitivity analysis, linear mixed-effects models with subject-specific random intercepts were fitted to evaluate the robustness of the repeated-measures findings. For secondary biometric and cardiovascular outcomes assessed at baseline and week 12, paired *t*-tests were applied after inspection of normality of paired differences. To control for multiple comparisons across secondary outcomes, *p*-values were adjusted using the Bonferroni method. Holm-adjusted *p*-values were also calculated as a sensitivity approach, yielding comparable results.

A two-sided *p*-value < 0.05 was considered statistically significant. Analyses were performed with STATA version 14.2. (StataCorp, College Station, TX, USA) and R software (version 4.4.3, R Foundation for Statistical Computing, Vienna, Austria).

### 2.7. Ethical Considerations

The study was conducted in accordance with the Declaration of Helsinki and approved by the Research Ethics Committee of Cádiz (Comité de Ética de la Investigación de Cádiz), protocol code NADACAR.OPS, registration number 55.22, date of approval: 4 July 2022. Written informed consent was obtained from all participants prior to inclusion.

### 2.8. Use of Generative AI

During manuscript preparation, the authors used ChatGPT (OpenAI, San Francisco, CA, USA; GPT-4–based large language model) for language editing and formatting according to journal guidelines. The authors reviewed and edited all AI-generated content and are responsible for the final text.

## 3. Results

### 3.1. Clinical and Demographic Characteristics

A total of 50 patients met the eligibility criteria and initiated participation in the study. One participant was excluded from the final analysis due to failure to complete the CR program at the study center. Consequently, 49 patients were included in the final analysis (mean age 65.6 years; 41% women). Baseline sociodemographic, clinical, and functional characteristics are summarized in Table 1. All had MDD with a HAM-D score ≥ 14 and at least one cardiovascular condition. The most frequent cardiopathies were ischemic cardiomyopathy (73%), arrhythmias (24%), and valvular disease (16%), with additional conditions including congenital anomalies and implanted cardiac devices. Most participants (62%) had two or more cardiovascular diagnoses. Non-cardiac comorbidities were present in 92% of patients—most commonly hypertension, neurological disorders, diabetes, and anxiety. Concomitant cardiovascular medication use was reported in 93%, most frequently statins (49%), beta-blockers (47%), and acetylsalicylic acid (43%).

All patients were treated with vortioxetine. At baseline, 96% initiated treatment with 10 mg/day and 4% with 15 mg/day. At the final visit, 74% remained on 10 mg/day, 12% were taking 15 mg/day, and 14% had been titrated to 20 mg/day. No treatment discontinuations occurred. Mild adverse effects were reported in 6.1% of patients (nausea in two cases, constipation in one), none requiring discontinuation or dose adjustment.

### 3.2. Depressive Symptoms and Functional Impairment

**Figure 1:**
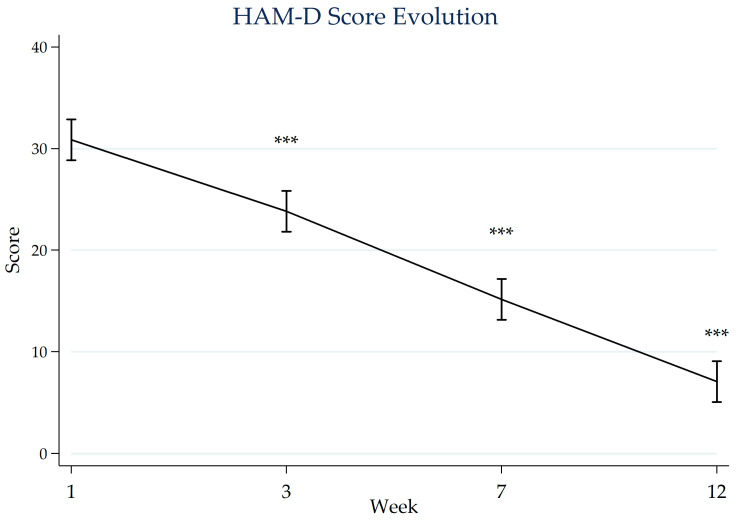
Mean HAM-D scores measured at baseline, week 3, week 7, and at the end of CR. Error bars represent 95% confidence intervals. Asterisks indicate statistical significance (***, *p* < 0.001) versus baseline based on Bonferroni-adjusted post hoc comparisons. CR: cardiac rehabilitation; HAM-D: Hamilton Depression Rating Scale.

**Figure 2:**
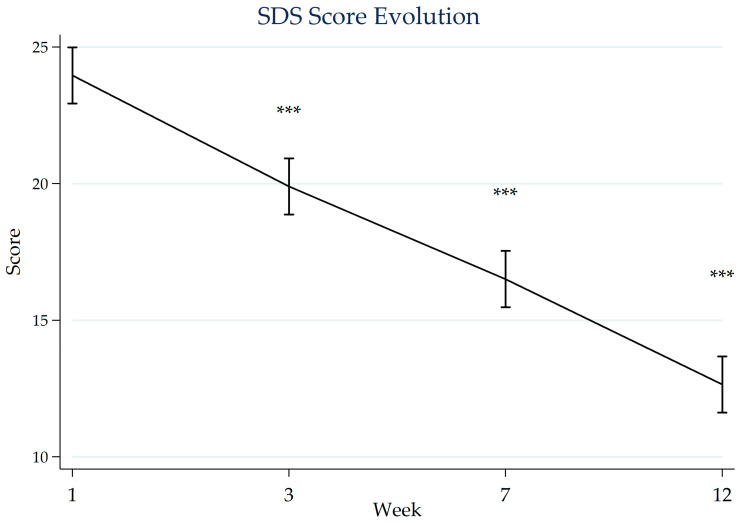
Mean SDS scores measured at baseline, week 3, week 7, and end of cardiac CR. Error bars represent the 95% confidence intervals. Asterisks indicate statistical significance (***, *p* < 0.001) versus baseline based on Bonferroni-adjusted post hoc comparisons. CR: cardiac rehabilitation; SDS: Sheehan Disability Scale.

Figure 1 and Figure 2 illustrate the progression of depressive symptoms and functional impairment over the 12-week follow-up period. Repeated-measures ANOVA identified statistically significant changes over time in both HAM-D scores (Greenhouse–Geisser corrected F(1.77, 85.01) = 277.52, *p* < 0.001) and SDS scores (F(1.50, 72.14) = 511.21, *p* < 0.001). Bonferroni-adjusted post hoc comparisons relative to baseline indicated lower mean scores at weeks 3, 7, and 12 (all *p* < 0.001). These findings were consistent in sensitivity analyses using linear mixed-effects models.

Mean HAM-D scores decreased over the follow-up period, from 30.9 (SD 9.3) at baseline to 23.8 (SD 8.3) at week 3, 15.2 (SD 5.8) at week 7, and 7.1 (SD 3.7) at week 12. At baseline, 76% of participants were classified as having severe depressive symptoms, 16% as moderate, and 8% as mild. At the final assessment, 39% were categorized as having mild symptoms and 61% as having no depressive symptoms.

Similarly, mean SDS scores decreased from 24.0 (SD 3.9) at baseline to 19.9 (SD 3.7) at week 3, 16.5 (SD 3.5) at week 7, and 12.7 (SD 3.5) at week 12. The distribution of functional impairment categories also changed over time, with baseline classifications of 80% severe and 20% moderate shifting to 2% severe, 69% moderate, and 29% with mild or no impairment at week 12.

### 3.3. Biometric and Cardiovascular Parameters

**Figure 3:**
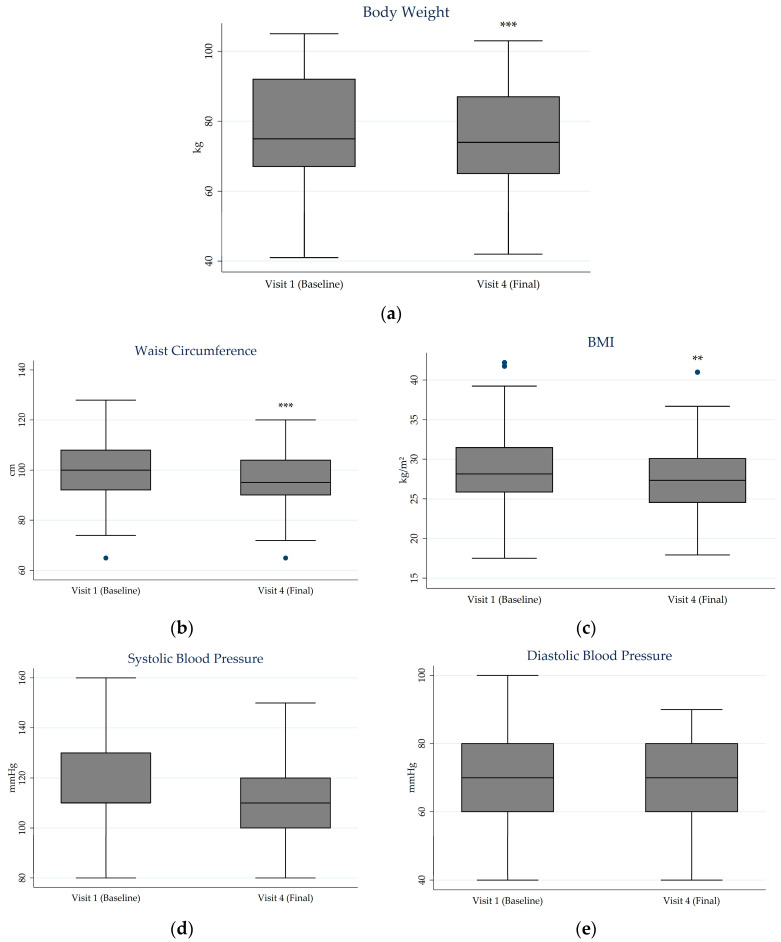
Boxplots of biometric variables at baseline and final visit: (**a**) body weight; (**b**) BMI; (**c**) waist circumference; (**d**) systolic blood pressure; (**e**) diastolic blood pressure. Each box represents the interquartile range (IQR), with the horizontal line indicating the median. Whiskers extend to values within 1.5 times the IQR, and points beyond this range are shown as potential outliers. Asterisks indicate statistical significance (**, *p* < 0.01; ***, *p* < 0.001). BMI: Body Mass Index.

Figure 3 presents box plot comparisons of biometric variables between the initial and final visits. Mean body weight decreased from 78.3 kg (SD 16.0) at baseline to 75.5 kg (SD 13.9) at week 12 (*p* < 0.001). Corresponding changes were observed in BMI, which decreased from 28.7 (SD 5.7) to 27.6 (SD 4.8) (*p* = 0.001), and in waist circumference, which decreased from 99.9 cm (SD 13.1) to 96.5 cm (SD 11.8) (*p* < 0.001). Blood pressure remained stable throughout the follow-up period with no significant differences for both systolic (from 116.5 mmHg (SD 17.7) to 112.9 mmHg (SD 14.3) (*p* = 0.297)) and diastolic pressures (from 70.1 mmHg (SD 12.2) to 68.0 mmHg (SD 10.4) (*p* = 0.787)).

**Figure 4:**
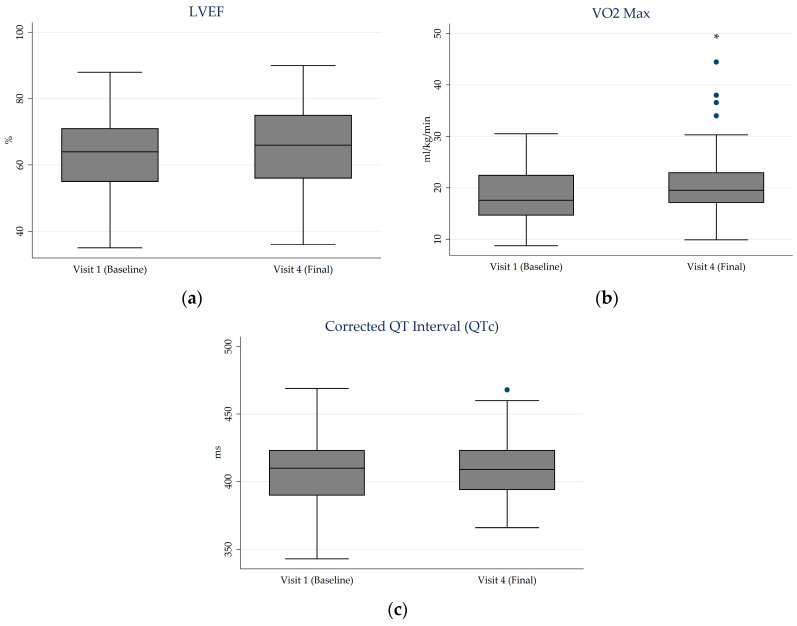
Boxplots of cardiac variables at baseline and final visit. (**a**) LVEF; (**b**) VO_2_ Max; (**c**) QTc. Each box represents the interquartile range (IQR), with the horizontal line indicating the median. Whiskers extend to values within 1.5 times the IQR, and points beyond this range are shown as potential outliers. Asterisks indicate statistical significance (*, *p* < 0.05). LVEF: Left Ventricular Ejection Fraction; VO_2_ max: peak oxygen uptake; QTc: corrected QT interval.

Figure 4 presents box plot comparisons of cardiac variables between the initial and final visits. Estimated VO_2_ max increased from 19.0 (SD 5.7) at baseline to 21.1 (SD 7.0) at week 12 (*p* = 0.030). Left ventricular ejection fraction values showed minimal change over the follow-up period, ranging from 63.2% (SD 13.1) at baseline to 65.8% (SD 13.5) at week 12 (*p* = 0.230). The corrected QT interval (QTc) remained stable, with mean values of 408.6 ms (SD 24.9) at baseline and 409.5 ms (SD 21.4) at week 12 (*p* = 1.000). No electrocardiographic abnormalities were reported during follow-up.

## 4. Discussion

This prospective observational study describes the longitudinal evolution of depressive symptoms, functional impairment, and selected cardiovascular and biometric parameters in patients with MDD and CVD undergoing CR while receiving vortioxetine as part of routine clinical care. Over the 12-week follow-up period, changes over time were observed in measures of depressive symptoms and functional status, alongside variations in selected anthropometric and cardiovascular parameters.

Importantly, these observations reflect real-world clinical conditions, in which pharmacological treatment for depression is implemented alongside structured CR. Accordingly, the results should be interpreted as describing symptom and functional trajectories over time rather than demonstrating causal treatment effects. The observed patterns underscore the complexity of managing depression in patients with CVD within multidisciplinary care settings.

In this context, the longitudinal patterns observed in the present study can be viewed alongside existing guideline recommendations that advocate an integrated approach to the management of depression in patients with CVD, encompassing pharmacological treatment, structured physical activity, and multidisciplinary CR [2,15,16,17,18].

Exercise constitutes a central component of CR programs, and prior research has documented associations between regular physical activity and changes in depressive symptoms, functional capacity, and quality of life in patients with CVD and comorbid depression [51,52,53]. In addition to aerobic modalities, previous studies have reported associations between resistance exercise and measures of muscular strength, functional performance, and depressive symptoms in cardiac populations [54].

The CR program implemented in the present cohort included systematically structured resistance training using a periodized model organized into sequential mesocycles. In line with prior literature, periodized resistance training has been associated with neuromuscular adaptations, exercise tolerance, and adherence in clinical populations [55,56]. Within this context, the structured nature of the exercise program may be relevant when considering the clinical trajectories observed in patients with depression, in whom fatigue, reduced motivation, and functional limitations can hinder engagement with less structured exercise approaches [54].

Several conceptual models described in the literature suggest that the clinical course of patients with comorbid depression and CVD may be influenced by the interaction of pharmacological treatment, structured exercise, and psychosocial support within multidisciplinary care settings [10,11,12,25]. Prior studies have reported associations between reductions in depressive symptom burden and changes in motivation, cognitive functioning, and emotional regulation, which may influence engagement and adherence to complex interventions such as cardiac rehabilitation programs [10,11,12,51,52,53]. In parallel, sustained participation in structured CR has been associated with changes in physical fitness, functional capacity, and quality of life in cardiac populations, including those with comorbid depressive symptoms [25,51,52,53,54]. Within this framework, the present study provides descriptive longitudinal data on how depressive symptoms, functional status, and selected cardiovascular parameters evolve over time in routine clinical practice.

Vortioxetine’s multimodal mechanism of action, combining serotonin reuptake inhibition with modulation of multiple serotonin receptors [57], has been associated not only with antidepressant effects but also with improvements in cognitive domains such as executive function, attention, and processing speed [36,38]. Although cognition was not formally assessed in this study, the observed functional trajectories are discussed in relation to previous trials reporting pro-cognitive effects in adult and elderly populations [58,59].

From a pathophysiological perspective, depression has been linked to autonomic nervous system dysregulation and inflammatory processes that adversely affect cardiovascular outcomes [60,61]. Unlike some other antidepressants, vortioxetine has not been shown to exacerbate autonomic dysfunction [62], and its indirect modulation of inflammatory pathways has been proposed in the literature as a potential contributor to its overall tolerability profile [63].

The present findings can be discussed in relation to previous evidence on vortioxetine in the treatment of MDD. A meta-analysis by Hughes et al. [39] reported improvements in depressive symptoms among patients undergoing CR, although effect sizes were modest. In contrast, earlier randomized trials such as MIND-IT [40] and ENRICHD [64], as well as broader meta-analyses [65,66], reported limited translation of mood improvement into cardiovascular or functional outcomes, highlighting the challenges of achieving integrated recovery in this population.

From a clinical standpoint, the results are discussed in relation to the feasibility of incorporating vortioxetine into integrated treatment strategies for patients with coexisting depression and CVD undergoing CR. Depression in cardiac populations has been associated with reduced adherence, increased morbidity, and poorer functional recovery, particularly among women [42]. In this context, the selection of antidepressant therapies that are both effective and well tolerated remains of particular relevance.

Safety remains an important consideration in this context. Several commonly used SSRIs, including citalopram and escitalopram, have been associated with QTc prolongation and hemodynamic effects in older or polymedicated patients [35,67]. In previous studies, vortioxetine has been described as having a limited impact on cardiovascular parameters. In the present cohort, blood pressure values and electrocardiographic measures remained stable over the follow-up period, in line with earlier reports [20].

### 4.1. Study Strengths

A key strength of this study is its real-world, prospective observational design, which reflects routine clinical practice in patients with CVD and comorbid MDD undergoing CR. This approach enhances the clinical relevance of the findings, particularly regarding feasibility, tolerability, and the longitudinal evolution of depressive symptoms, functional impairment, and intermediate cardiovascular parameters in a complex and highly prevalent patient population.

Another notable strength is the comprehensive and structured nature of the CR program. Unlike more conventional rehabilitation protocols that predominantly emphasize aerobic exercise, the program evaluated in this study incorporated systematically planned resistance training using a periodized model with predefined mesocycles and progressive load adjustment. This structured approach may have contributed to improvements in functional capacity and adherence, and represents an innovative aspect of the rehabilitation strategy within a real-world clinical setting.

The study also benefits from the use of validated clinical instruments for the assessment of depressive symptoms and functional impairment, as well as standardized and consistently applied cardiovascular and anthropometric measurements. All cardiovascular assessments were performed and interpreted by the same cardiologist, reducing interobserver variability. In addition, follow-up assessments were conducted at multiple time points, allowing for a detailed evaluation of symptom trajectories over the rehabilitation period.

Finally, the integration of psychiatric treatment within a multidisciplinary CR framework represents an important clinical strength, highlighting the value of coordinated care between cardiology, rehabilitation, and mental health services for patients with CVD and comorbid depression.

Overall, the present findings highlight the complexity of treating patients with coexisting CVD and MDD within routine clinical practice. The observed improvements in mood, functional status, and selected cardiovascular parameters appear to reflect the combined effects of optimized antidepressant therapy and a structured, multidisciplinary CR program that includes systematically planned exercise. While causal inferences cannot be drawn, the results underscore the potential clinical value of integrated care models addressing both mental and physical health dimensions in this high-risk population. In line with the lack of consensus highlighted in current guidelines regarding the optimal management of depressive symptoms within CR programs, this study provides exploratory real-world data on the feasibility and safety of an integrated pharmacological and exercise-based approach in routine clinical practice.

### 4.2. Study Limitations

Several limitations of this study should be acknowledged. First, the observational, single-arm design without a control or comparator group precludes causal inference regarding the effects of vortioxetine, CR, or their relative contribution to the observed improvements. Consequently, changes in depressive symptoms, functional status, and cardiovascular parameters cannot be attributed to a specific intervention, and the findings should be interpreted as associative rather than causal.

Second, all participants were enrolled in a structured CR program concurrently with antidepressant treatment. Exercise, lifestyle modification, and multidisciplinary support are well-established contributors to improvements in functional capacity and mental health, and therefore represent important potential confounding factors. Although this reflects real-world clinical practice, it limits the ability to disentangle the independent effects of pharmacological treatment from those of rehabilitation.

Third, the study was conducted at a single center with a modest sample size, which may limit the generalizability of the findings. The absence of randomization and the reliance on routine clinical prescribing decisions may also introduce selection bias. In addition, the relatively short follow-up period restricts conclusions regarding the long-term sustainability of the observed improvements.

From a methodological perspective, although validated clinical scales and standardized measurement procedures were used, some clinical variables—such as NYHA functional class—were not systematically recorded. Furthermore, while repeated-measures analyses were supported by sensitivity analyses using mixed-effects models, the sample size limits the exploration of subgroup effects or more complex multivariable adjustments.

Finally, the structured and periodized resistance training approach used in the rehabilitation program differs from more conventional CR protocols. While this may represent a strength in terms of innovation, it also limits direct comparability with other studies and may reduce external validity.

## 5. Conclusions

In a real-world cardiac rehabilitation setting, patients with major depressive disorder and comorbid cardiovascular disease who were undergoing structured cardiac rehabilitation while receiving vortioxetine as part of routine clinical care showed consistent longitudinal changes in depressive symptoms, functional status, and selected cardiovascular and biometric parameters over a 12-week period. These findings provide descriptive evidence on how psychological, functional, and cardiovascular dimensions may evolve concurrently in a clinically complex population treated with vortioxetine within an integrated, multidisciplinary rehabilitation framework. The absence of relevant electrocardiographic or hemodynamic alterations during follow-up further supports the clinical feasibility of this integrated approach in everyday practice. Rather than establishing treatment effects, this study contributes hypothesis-generating real-world data that help contextualize the use of vortioxetine in patients with comorbid depression and cardiovascular disease participating in cardiac rehabilitation programs, and underline the importance of jointly considering mental health, physical function, and cardiovascular parameters when designing future controlled studies in this population.

## Figures and Tables

**Table 1:** Sociodemographic, clinical, and functional characteristics of the study population at baseline.

	Variable	Mean (SD)/n (%)
Sociodemographic	Age; Mean (SD)	65.6 (9.6)
	Men	29 (59%)
	Women	20 (41%)
Depression	HAM-D Score (Baseline); Mean (SD)	30.9 (9.3)
	Mild Depression	4 (8%)
	Moderate Depression	8 (16%)
	Severe Depression	37 (76%)
Functionality	SDS Score (Baseline); Mean (SD)	24.0 (SD 3.9)
	Mild functional impairment	0 (0%)
	Moderate functional impairment	10 (20%)
	Severe functional impairment	39 (80%)
Heart disease	Ischemic heart disease	36 (73%)
	Valvulopathy	8 (16%)
	Arrhythmias	12 (24%)
	Heart failure	6 (12%)
	Congenital cardiomyopathy	6 (12%)
	Implanted devices	8 (16%)
	Avg. number of cardiac conditions per person; Mean (SD)	2 (0.84)
Comorbidities	Diabetes	3 (6%)
	Renal failure	2 (4%)
	Neurological disorder	3 (6%)
	Sleep apnea	2 (4%)
	Anxiety	2 (4%)
	Dyslipidemia	1 (2%)
	Hypertension	4 (8%)
	Respiratory	2 (4%)
	Other comorbidities	6 (12%)
	Avg. number of comorbidities per person; Mean (SD)	2 (1.18)
Cardiology treatments	ACEIs	9 (18%)
	ARBs	8 (16%)
	Beta blockers	23 (47%)
	Statins	24 (49%)
	Antianginal agents	16 (33%)
	ASA	21 (43%)
	Anticoagulants	14 (29%)
	Diuretics	8 (16%)
	Avg. number of concomitant drugs	3 (1.32)
Adverse drug reactions	Total adverse effects	3 (6%)
	Nausea	2 (4%)
	Constipation	1 (2%)

Values are presented as mean (standard deviation) or frequency (percentage). Functional impairment is based on the Sheehan Disability Scale (SDS), and depressive symptoms on the Hamilton Depression Rating Scale (HAM-D). ACE: Angiotensin-Converting Enzyme Inhibitors; ASA: Acetylsalicylic Acid; ARBs: Angiotensin II Receptor Blockers; n: frequency; SD: Standard Deviation.

## Data Availability

The data presented in this study are available on reasonable request from the corresponding author. The data are not publicly available due to privacy restrictions.

## Abbreviations

The following abbreviations are used in this manuscript:

MDD	Major Depressive Disorder
CVD	Cardiovascular Disease
CR	Cardiac Rehabilitation
HAM-D	Hamilton Depression Rating Scale
SDS	Sheehan Disability Scale
LVEF	Left Ventricular Ejection Fraction
BMI	Body Mass Index
VO_2_ max	Peak Oxygen Uptake
QTc	Corrected QT Interval
